# Thermal stimulation with meridian points for enhanced recovery after spine surgery: A PRISMA-compliant protocol for network meta-analysis of randomized controlled trials

**DOI:** 10.1097/MD.0000000000033909

**Published:** 2023-06-02

**Authors:** Jung-Hyun Kim, Bonhyuk Goo, Byung-Kwan Seo

**Affiliations:** a Department of Acupuncture & Moxibustion, Kyung Hee University Hospital at Gangdong, Seoul, Republic of Korea; b Department of Acupuncture & Moxibustion Medicine, Kyung Hee University College of Korean Medicine, Kyung Hee University Hospital at Gangdong, Seoul, Republic of Korea.

**Keywords:** enhanced recovery, ERAS, integrative medicine, moxibustion, network meta-analysis protocol, spine surgery, thermal stimulation on meridian points

## Abstract

Enhanced recovery after surgery programs are widely known as a far-reaching approach to help patients with surgeries recover rapidly by giving optimized care. However, especially in the spine medicinal domain, relevant studies are scarce. In order to manage proper recovery after surgery, practitioners need to take a comprehensive look at the suppression of pain, strategies for improving the results of surgery, improving the quality of life, and mental support of patients. To this end, a recovery-enhancing program through integrated medical technology which covers both conventional and Korean medicine is highly required. We want to develop an integrated medical program that covers a wide range of medicinal domains for the early recovery of patients after spinal surgery. The aim of the present study is to check out which thermal stimulation with meridian points are more effective than others to enhance recovery after spinal surgery.

**Methods:**

The following online databases will be retrieved in the present study: PubMed; Scopus; the Cochrane Central Register of Controlled Trials; Ovid MEDLINE; Ovid EMBASE; Chinese Biomedical Literature Database; China National Knowledge Infrastructure; and Chinese Scientific Journal Database (VIP database). We will independently classify articles and will encapsulate characteristics of the study components. Primary outcomes will be categorized into visual analog scale, Tolerance to liquid and solid diet, postoperative hospitalization period, and quality of life. Secondary outcomes will be analyzed based on the study findings.

**Results and Conclusion:**

The results of this study will be submitted to a peer-reviewed journal for publication. Furthermore, the outcomes of this study would afford the documentation of whether thermal stimulation on meridian points can be effective for enhanced recovery after spine surgery.

## 1. Introduction

The notion of enhanced recovery after surgery (ERAS), also called expeditious or swift recovery, was first suggested by Henrik Kehlet.^[1]^ By him, an evidence-based proposition to treat was devised to arrange patients for, and cut down the influence of surgery, allowing them to bounce back more rapidly. ERAS aims to curtail the surgical stress response to diminish complications after the operation and enhance surgical outcomes and functional improvement after the operation.^[2,3]^ ERAS has been proven to decrease the possibility of further complications,^[4–10]^ cut down the length of stay,^[4,6,7,9,11]^ and increase pain scores after the operation.^[8]^

Spinal surgery speaks for a typically invasive modality with a sustained recovery phase that often demands reconstruction and comprehensive pain management after surgery. Considering the gains of ERAS to cut down complications and recover physiologic and psychological status, its embodiment or inclusion into spinal surgery shows a natural transition.^[12]^

Recently, researchers have been paying attention to complementary alternative medical treatments, besides conventional medical approaches, for early recovery after spinal surgery. Representative approaches to complementary alternative medicine, which are widely used in East Asia, such as Korean medicine and traditional Chinese medicine, include physical stimulation with meridian points.^[13]^

Physical stimulation with meridian points can be widely categorized with acupuncture; which is stimulation by needling and thermal stimulation which utilizes heat or radiant energy. Vastly known as a key modality in complementary and alternative medicine, acupuncture generally represents analgesic results through para-spinal and peripheral procedures. Moreover, it may help to avert preoperative anxiety and diminish discomfort as well as complications after surgery.^[14,15]^

Thermal stimulation with meridian points is derived from the traditional form of moxibustion therapies in traditional Chinese medicine. Although moxibustion shows a direct or indirect application of Artemisia argyi at acupoints or other specific parts, thermal stimulation used a wider variety of heat sources other than Artemisia argyi.^[16]^ Considering that the safety of thermal stimulation has been verified through numerous studies,^[17]^ and that it acts similarly to acupuncture in terms of stimulating the meridian point,^[18]^ it is possible to infer that the application of thermal modalities with meridian points will facilitate postoperative recovery.

However, despite the above rationale and a number of clinical insights, no scientific approach has revealed which thermal stimulation varying in depth, time, and material with application promotes postoperative recovery. The researchers planned this present study to answer this question.

## 2. Objectives

This article is aimed at evaluating the comparative effectiveness of various thermal stimulation with meridian points in promoting enhanced recovery after spine surgery.

• P: patients who had undergone spinal surgery in the past 1 year.
• I: thermal stimulation for specific meridian points based on the meridian theory in traditional Chinese medicine.
• C: conventional medical treatment, sham stimulation with heat energy, and conventional acupuncture groups.
• O: visual analog scale, tolerance to liquid and solid diet, postoperative hospitalization period, quality of life (EuroQol-5 dimension).

## 3. Material and methods

### 3.1. Study enrollment

The protocol of this network meta-analysis (NMA) is enrolled with PROSPERO CRD42023407424 (https://www.crd.york.ac.uk/prospero/display_record.php?RecordID=407424). Additional ethical approval was not necessary provided that the present study is not classified as a prospective study.

### 3.2. Ethical approval

Ethical approval was not needed. Individual patients’ data will not be included in the paper.

### 3.3. Design of the study

We will proceed with an NMA regarding the registration guidelines of the Preferred Reporting Items for Systematic Reviews and Meta-Analyses statement.^[19]^

### 3.4. Criteria of eligibility

#### 3.4.1. Participants.

All papers that presented a clinical trial in which patients who underwent spinal surgery were treated with any kind of thermal stimulation on meridian points were included. There would be no restrictions on the age and gender of the target patient population, nor on surgical techniques. Also, there will be no restrictions on what kind of symptoms aims to improve, such as digestive functions, pain relief, and insomnia after spinal surgery.

#### 3.4.2. Class of interventions.

Suitable modalities contain all types of thermal stimulation on meridian points used for early recovery in postoperative care procedures. There will be no restrictions on the types of thermal stimulation such as indirect or direct mugwort and electric moxibustion, and applied temperature. Regarding wide variety of modalities in complementary and alternative medical fields, thunder fire stimulation, needle warming moxibustion, taiyi miraculous moxa roll, and suspended moxibustion are considered for inclusion. In the case of a combination of conventional treatment, medical medication, etc., and if thermal stimulation with meridian points is included, it will be included.

#### 3.4.3. Type of articles.

Authors will contain controlled clinical trials distributed until February 2023 that assess the effectiveness of thermal stimulating modalities on meridian points specifically devised to manage subjects who underwent spinal surgery.

Authors shall rule out quasi-experimental, single-arm pre-post, or historically controlled articles. Trials testing other modalities, such as dry acupuncture, needling, or acupoint injections were ruled out. Gray literature, dissertations, and articles with abstracts were incorporated provided they enclosed adequate components. There would be no language restraint on reviewing articles.

#### 3.4.4. Type of comparator(s) or control.

The comparative mediations would be sham heat stimulation and sham moxibustion, with no treatment, herbal decoction, conventional medication, or placebo in the possible control group.

#### 3.4.5. Outcome measures.

Authors are mainly attentive to outcomes that assess one of the following 4 main domains:

1. Visual analog scale.
2. Tolerance to liquid and solid diet.
3. Postoperative hospitalization period.
4. Quality of life.

Secondary outcomes will be analyzed banking on findings of retrieved articles; expected possibilities contain concomitant promotion of daily activities or adverse events.

#### 3.4.6. Language.

When considering the languages of the regions where thermal stimulation and moxibustion are mainly utilized for treatment, languages written in English, Korean, and Chinese will be selected. Other languages will be excluded from the retrieval process due to the scarcity of those available.

### 3.5. Search strategy

We will devise and conduct a search plan using approaches suggested by the Institute of Medicine and will involve several electronic databases: PubMed; Scopus; the Cochrane Central Register of Controlled Trials; Ovid MEDLINE; Ovid EMBASE; Chinese Biomedical Literature Database; China National Knowledge Infrastructure; and Chinese Scientific Journal Database (VIP database). To boost the particularity and expediency of the retrieval process, authors would concentrate on Medical Subject Headings such as “moxibustion,” “thermal stimulation” and “meridian points” along with text results of terms such as “ERAS,” “Spine surgery,” “Surgery,” “After surgery” and “Early recovery” (which is a term utilized by some in referencing an identical assembly). Search strategy demonstrated with MEDINE via Pubmed is shown in Table 1.

**Table 1:** Search strategy for the MEDLINE via PubMed.

#1. “ERAS”[MeSH] OR “spine surgery”[MeSH] OR “enhanced recovery”[MeSH] OR “eary recovery”[MeSH] OR “after surgery”[MeSH] OR “ERAS”[Title/abstract] OR “spine surgery”[Title/abstract] OR “enhanced recovery” [Title/abstract] OR “early recovery”[Title/abstract] OR “after surgery”[Title/abstract]
#2. “thermal stimulation”[MeSH] OR “heat stimulation”[MeSH] OR “moxibustion”[MeSH] OR “thermal stimulation”[Title/abstract] OR “heat stimulation”[Title/abstract] OR “moxibustion” [Title/abstract]
#3. #1 AND #2

ERAS = enhanced recovery after surgery, MeSH = Medical Subject Headings.

The notion of therapy will be consigned mainly using text words such as “thermal stimulation on meridian points,” “moxibustion,” “heating stimulation,” “Thunder fire,” “taiyi miraculous moxa roll,” and “needle warming.” The original retrieval plan would be reinforced by manual retrieval of the reference lists of suitable papers and by connecting experts in the domain to pinpoint any missing, not-completed, or not-distributed articles. Additionally, researchers will investigate for reviews on the theme and peruse their reference lists to find out possibly suitable articles that may have been missed through other approaches. Lastly, clinical trial registries will be retrieved to determine concluded and in-progress papers; if not selected through other approaches, the authors of original papers will be contacted for details regarding study components.

## 4. Analysis

### 4.1. Selection of the articles

**Figure 1.**
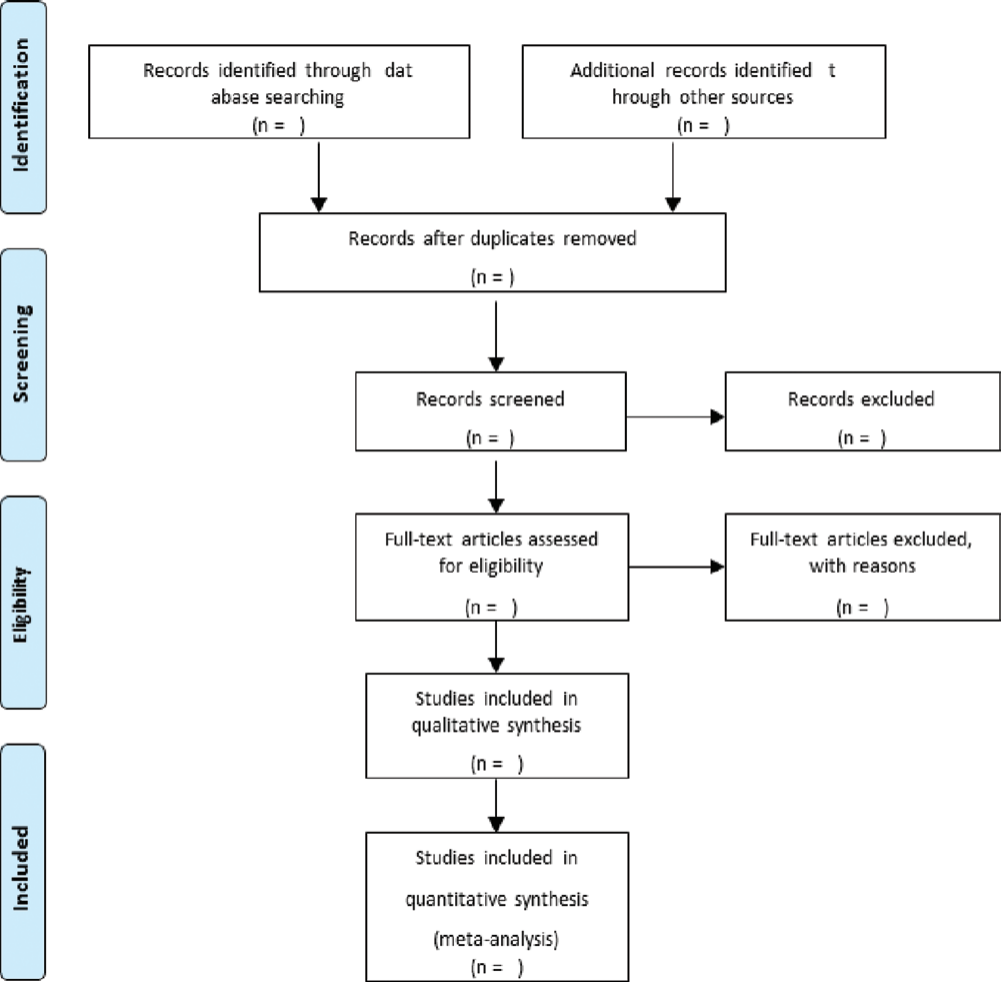
PRISMA-P flow diagram. PRISMA-P = Preferred Reporting Items for Systematic Reviews and Meta-Analyses-Protocol.

Researchers will transmit retrieval outcomes into software for systematic review (SR) (DistillerSR, Evidence Partners, Ottawa, ON). In the first round of seclusion, titles, and abstracts would be reviewed for inclusion. Following abstract seclusion, suitability will be examined through full-text retrieval. In advance of both abstract review and full-text screening, reviewers will proceed with drills to secure a primary apperception of the domain and aim of the review. Apprehension of inclusion and exclusion criteria will be examined through appraisal of a small number of articles. Early-discovered papers, with no time frame and language restrictions on various search engines, will be first ruled out by checking the redundancy of endnotes and by identifying the researchers themselves as countries, authors, titles, and contribution journals. Subsequently, the title and abstract review will exclude studies that do not conform to the topic (the early recovery from spine surgery or the effects of thermal stimulation with meridian points on recovery), studies that do not conform to the type of research (randomized clinical trial [RCT], SR that deals with RCT), and studies that do not conform to language conditions (English, Korean, and Chinese). Finally, we will review the full text and similarly rule out studies that do not conform to the subject, form, and language of the study and finalize the remaining studies. If there are studies that cannot locate full text, researchers will contact the corresponding journals and authors to request full text and nonetheless exclude those that fail to obtain the full text. The summarized retrieval procedure can be referred to in Figure 1.^[20]^

### 4.2. Management of data

The articles will be handled within the reference managing software of EndNote X9 (Endnote X9, Clarivate Analytics, CA). De-duplication will be expedited by EndNote also.

### 4.3. Extraction on data

Working independently with authors, data extractors will compile initial data from the contained trials with a web-based software (DistillerSR). The extricated data will contain characteristics of patients, outcomes measured and instruments utilized, factors relevant to study quality, and risk of bias assessment. Disparities in data assortment would be arbitrated with a consensus-making procedure. If data shown in the articles are vague, lacking, or presented in a form that is difficult to extract reliably, the authors of the corresponding study will be connected for offers with clarification. When the data extrication procedure is complete, the authors of the studies will be connected to secure the fullness of data elicitation. Additionally, the authors of included articles would be asked if they are aware of any further studies that they suppose would be suitable for our retrieval process.

Contacting with original authors would be started using e-mail to the corresponding author of the study. If no e-mail address is shown, a search on the internet will provide us to identify an available e-mail address. If e-mails for the corresponding author are not available, corresponding authors will be connected by telephone number or fax.

Original authors will be given 2 weeks to reply to e-mails, at which time a checking email will be delivered; if no answer is received after an additional 2 weeks, a call with a telephone number will be made to try to contact the author. If the author could not be contacted through a given telephone number, the author was encouraged to answer the telephone or email via fax number presented on the internet or already published papers.

### 4.4. Risk of bias assessment

Originally, the authors will utilize the Cochrane Collaboration’s risk of bias tool to assess the methodological quality of retrieved studies.^[20]^ If the corresponding article is categorized as a non-RCT, the risk of bias will be examined using RoBINS-I. RoBINS-I represents the risk of bias evaluation tool used in nonrandomized clinical studies.^[21]^

The risk of bias with corresponding articles is classified into 3 grades; high, low, and unclear. Included studies will be checked twice by reviewers working individually. Any discrepancies would be concluded by consensus establishing procedure. If consensus is unable to be made, a third reviewer will intervene. Items in the risk of bias evaluation will contain randomization, quality of randomization (any important imbalances at baseline), allocation concealment, level(s) of blinding/masking, losses to follow-up, intention to treat analysis, handling of missing data, and funding sources.

### 4.5. Measures of the intervention effect

We will measure the therapeutic effect of thermal stimulation on meridian points as the mean difference or standardized mean difference (SMD). Mean difference with 95% confidence interval (CI) will be utilized to examine the effect of continuous data. SMD will be used to assess the study outcome if different outcome scales or tools were used in articles. The risk ratio with 95% CI will be utilized to examine the effect of binary data.

### 4.6. Data analysis

#### 4.6.1. Procedure of analysis.

To compare active intervention with the same mediation with different controls, the authors will proceed with a pairwise meta-analysis. Projected Bayesian NMA method will assess the related outcomes by contrasting thermal stimulation on meridian points with controlled conditions.

#### 4.6.2. Pairwise meta-analyses.

Authors will conduct pairwise meta-analyses for each pairwise comparison to calculate the odds ratio for dichotomous outcomes or SMD for continuous outcomes, both with 95% CIs. The heterogeneity of each pairwise comparison will be calculated by *x*^2^ test, a fixed effect model was selected for meta-analysis. If statistical heterogeneity is suggested among the outcomes (*P* < .1, *I*^2^ > 50%), the source of heterogeneity needs to be evaluated. Among the outcomes of various articles, when heterogeneity is exceeded, sensitivity analysis and descriptive analysis will be considered to handle the heterogeneity.

#### 4.6.3. Missing data management.

To acquire the lacking data, we would try to contact the corresponding authors or primary authors. If there is no response to our contact trial, we will include only the available data.

#### 4.6.4. Reporting bias assessment.

We will assess the reporting bias using funnel plot if the number of studies included in the analysis would be adequate.

#### 4.6.5. Inconsistency assessment.

The disparity between direct and indirect evidence can be investigated by the Consistency hypothesis. In the present article, the Consistency hypothesis plays a key role in NMA. When a loop is settled among mediations, authors can demonstrate *Z* test to evaluate the inconsistency.

#### 4.6.6. Heterogeneity evaluation and subgroup analysis.

We will examine heterogeneity across studies using the *I*^2^ value derived from *X*^2^ test. The *I*^2^ score shall be appraised to evaluate discrepancies in the outcomes of the contained studies. To assess substantial heterogeneity, we will explore possible causes by conducting sensitivity and subgroup analyses.

#### 4.6.7. Assessing the quality of evidence.

The quality of evidence will be thoroughly considered by 2 independent authors to review preliminary recommendations. Authors will consider the Grading of Recommendations Assessment, Development and Evaluation Grid instrument to check each recommendation to group it into one of the following 5 options, from “strong no recommendation” to “strong recommendation.” This classification into 5 categories aims to reach a better consensus. To investigate the quality of evidence, professionals with relevant fields including the authors of the present article will be invited and gathered as an expert panel. If 3/4 of the experts reach a consensus on an option, there is agreement on the specific recommendation. If not, the issue goes to the next Delphi process again. To sum up, the authors will investigate the quality of intervention effect estimates from NMA by the following steps.

1. List treatment estimates with thermal stimulation on meridian points from each comparison of the network.
2. Evaluate the quality of each intervention effect estimate.
3. Present the NMA estimate for each comparison of the evidence
4. Rate the quality of each NMA effect estimate.

## 5. Discussion

Spinal surgery massively affects the quality of life of individual subjects and the satisfaction of postoperative care, depending on how long it takes to restore daily function after surgery. This has led to a number of studies on programs for enhanced recovery based on additional medical treatments before, during, and after surgery. Despite surgical treatment being one of the conventional medical techniques, complementary and alternative approaches including Korean medicine and traditional Chinese medicine could help manage and improve the various discomfort symptoms that patients complain about after surgery. Spinal surgery is gradually increasing with age, and the development of an integrated medical system for the early recovery of the patient is of great significance. The application of integrated medical care in the clinical field requires mutual understanding and consultation between various medical systems, and the establishment of clear evidence for each treatment must precede. In this regard, the SR handling this issue will perform a crucial role in laying the groundwork for the utility of thermal stimulation on meridian points among a number of Korean medical treatments applicable to spinal surgery patients.

## Acknowledgements

The authors would like to express our deepest gratitude to Editage for the English editing service on this manuscript.

## Author contributions

**Conceptualization:** Jung-Hyun Kim.

**Data curation:** Jung-Hyun Kim.

**Formal analysis:** Jung-Hyun Kim.

**Funding acquisition:** Byung-Kwan Seo.

**Investigation:** Jung-Hyun Kim.

**Methodology:** Jung-Hyun Kim.

**Project administration:** Bonhyuk Goo.

**Resources:** Bonhyuk Goo.

**Software:** Bonhyuk Goo.

**Supervision:** Byung-Kwan Seo.

**Validation:** Bonhyuk Goo.

**Visualization:** Bonhyuk Goo.

**Writing – original draft:** Jung-Hyun Kim.

**Writing – review & editing:** Jung-Hyun Kim.
